# l-Fucose ameliorates high-fat diet-induced obesity and hepatic steatosis in mice

**DOI:** 10.1186/s12967-018-1718-x

**Published:** 2018-12-07

**Authors:** Guangyan Wu, Mengwei Niu, Wenli Tang, Jingjuan Hu, Guoquan Wei, Zhanke He, Yangping Chen, Yong Jiang, Peng Chen

**Affiliations:** 1 Department of Pathophysiology, Guangdong Provincial Key Laboratory of Proteomics, School of Basic Medical Sciences, Southern Medical University, Guangzhou, China; 2 State Key Laboratory of Organ Failure Research, Southern Medical University, Guangzhou, China; 3 Division of Laboratory Medicine, Zhujiang Hospital, Southern Medical University, Guangzhou, China

**Keywords:** l-Fucose, Obesity, Hepatic steatosis, High-fat diet, Gut microbiota

## Abstract

**Background:**

l-Fucose (Fuc), a six-deoxy hexose monosaccharide, is present endogenously in humans and animals and has a wide range of biological functions. In the present study, we aimed to examine the effect of Fuc on obesity and hepatic steatosis in mice fed a high-fat diet (HFD).

**Methods:**

C57BL/6 mice were fed a normal chow (NC) or HFD for 18 weeks to induce obesity and fatty liver. Fuc was administered intragastrically from the 8th week to the end of the experiment (18 weeks).

**Results:**

Metagenomic analysis showed that HFD altered the genomic profile of gut microbiota in the mice; specifically, expression of alpha-l-fucosidase, the gene responsible for Fuc generation, was markedly reduced in the HFD group compared with that in the NC group. Fuc treatment decreased body weight gain, fat accumulation, and hepatic triglyceride elevation in HFD-fed mice. In addition, Fuc decreased the levels of endotoxin-producing bacteria of the *Desulfovibrionaceae* family and restored HFD-induced enteric dysbiosis at both compositional and functional levels.

**Conclusion:**

Our findings suggest that Fuc might be a novel strategy to treat HFD-induced obesity and fatty liver.

## Background

The epidemic of obesity and obesity-associated liver diseases in particular are a major cause of death worldwide and their prevalence is at unprecedentedly high levels [1, 2]. Notably, it has been widely shown that gut microbiota is associated with development of liver disease in mice, including acetaminophen-induced acute liver injury [3, 4], ethanol-induced liver injury [5, 6], and high-fat diet (HFD)-induced non-alcoholic fatty liver disease (NAFLD) [7, 8]. NAFLD, which occurs along with obesity, is the major cause of liver disease currently [9]. NAFLD ranges from simple steatosis to non-alcoholic steatohepatitis (NASH), fibrosis, and carcinoma [10]. However, details of NAFLD pathogenesis are still unclear and treatment approaches are limited.

Studies conducted over the last decade have shown that HFD feeding leads to accumulation of adipose tissue and NAFLD development, which is related to compositional and functional dysbiosis of gut microbiota [11, 12]. Increasing evidence indicates that gut microbiota plays an important role in bidirectional communication between the gut and liver. For example, the significance of the protective role of prebiotics and probiotics in restoring disrupted gut microbiota has been widely demonstrated. Further, a considerable number of studies have revealed that both gut microbiota and their metabolites are crucial to maintaining host homeostasis [8, 13]. Although the modulation of HFD-induced abnormalities by gut microbiota is well-established, the detailed associations are not completely clear. In the present study, through using metagenomic analysis, we found that expression of the alpha-l-fucosidase gene (*fuca*), which is responsible for l-Fucose (Fuc) generation, was markedly decreased at the genomic level in HFD treated gut microbiota in mice, indicating that Fuc, a six-deoxy hexose monosaccharide, may play an important role in HFD-induced abnormalities. Some studies have suggested that rodents are unable to metabolize Fuc for energy [14, 15]. It has also been reported that Fuc and fucose-rich polysaccharides efficiently stimulate elastin biosynthesis and deposition [16]. However, little is known about the relationship of Fuc with HFD-associated liver disease or intestinal eubiosis in mice. We therefore aimed to evaluate the effects of intragastric administration of Fuc on HFD-induced obesity, hepatic steatosis, and insulin resistance in a mouse model; we also investigated the relationship between Fuc and enteric eubiosis during the development of obesity and fatty liver.

## Materials and methods

### Animals and human samples

Five-to-six-week-old male specific pathogen-free C57BL/6 mice were used in the study. All mice were randomly divided into the following three groups; (1) normal chow (NC), fed a low calorie fat diet (3.85 kcal/g, #D12450-B, Guangdong medical laboratory animal center); (2) high-fat diet (HFD), fed a high-fat diet (5.24 kcal/g, #D12492, Guangdong medical laboratory animal center); and (3) high-fat diet + Fuc (HFD + Fuc), fed a high-fat diet and administered intragastrically with Fuc (0.3 g/kg, F11093, Aladdin^@^) once a day from the 8th week until the end of the experiment; mice in the NC and HFD group were administered intragastrically with an identical dose of sterile ddH_2_O. All animals had access to water and food ad libitum, and their body weight was monitored weekly until the experiment was terminated at 18 weeks. All the mice were kept under a 12 h/12 h light/dark cycle with lights on at 8:00 AM and off at 8:00 PM. All mice were anesthetized and sacrificed after 18 weeks. The animal care and study protocols were in accordance with the guidelines of the Institutional Animal Care and Use Committee at Southern Medical University.

Stools from NAFLD patients (ultrasound approved) and age- and gender- matched healthy controls were collected. All individuals gave informed consent, and the study was approved by the Ethical Committee of Southern Medical University.

### DNA extraction

Cecal contents were collected after the mice were anesthetized, and were frozen immediately in liquid nitrogen and stored at − 80 °C. Microbial DNA was extracted from cecal contents as previously described [5, 17, 18]. Briefly, the cecal contents were resuspended separately in phosphate buffer solution (PBS) (pH 7.4) containing 0.5% Tween 20 and vortexed gently followed by a − 80 °C/60 °C cycle three times to disrupt bacterial membranes. DNA extraction was performed using the phenol–chloroform method.

### Metagenomic analysis

Microbial DNA was extracted from the cecal contents using the protocol described above. All samples were paired-end sequenced on the Illumina platform, the reads aligned to the race genome were removed after quality control, and the remaining high-quality reads were used for further analysis. Gene abundance was normalized to reads. We classified the predicted genes by aligning them to genes in the Kyoto Encyclopedia of Genes and Genomes (KEGG) pathways [19]. The KEGG orthology abundance was then calculated by summing the abundance of genes annotated to the same feature [20].

## 16S rRNA gene sequencing

DNA extracted from the cecal contents was used to amplify the highly conserved variable region 4 (V4) of the bacterial 16S rRNA gene using polymerase chain reaction (PCR). The V4-16S rRNA gene was amplified by the following barcoded primers (V4F, 5′-GTGTGYCAGCMGCCGCGGTAA-3′ and V4R, 5′-CCGGACTACNVGGGTWTCTAAT-3′), according to the preparation instructions for Illumina Hiseq PE250. Raw reads were first screened for low quality bases and short read lengths, and then demultiplexed and clustered into operational taxonomic units (OTUs) (97% similarity) [21]. Alpha diversity and beta diversity were calculated using QIIME. To account for uneven sampling depth, the data were also rarefied to the minimum sampling depth of 16,900 sequences. Principal coordinate analysis (PCoA) plots were constructed and visualized with R (v3.2.2) and ade4 R packages, then tested for significance with Adonis (999 permutations).

### Functional pathways prediction analysis

Predicted functions of the gut microbiota were inferred for each cecal content sample using the Phylogenetic Investigation of Communities by Reconstruction of Unobserved States (PICRUSt). The main oscillatory functional pathways were plotted in a heatmap. The heatmap represented the main oscillating functional KEGG pathways at the class level with the values centered and scaled along rows.

### Glucose tolerance test and insulin tolerance test

Glucose tolerance test (GTT) was performed at 15 weeks, while insulin tolerance test (ITT) was performed at 16 weeks [22]. For the GTT, the mice were transferred into a cage with fresh bedding and had free access to water; following fasting for 16 h, 1 g/kg glucose was administered (i.p). Blood glucose concentration (OneTouch Ultra^**®**^ test strips) was measured at 0 min, 30 min, 60 min, 90 min, and 120 min after glucose administration. For the ITT, the mice were transferred into a cage with fresh bedding and had free access to water; following fasting for 6 h, 0.35 U/kg insulin was administered (i.p). Blood glucose concentration (OneTouch Ultra^**®**^ test strips) was monitored at 0 min, 30 min, 60 min, 90 min, and 120 min after insulin administration.

### Hematoxylin and eosin staining

The livers and epididymal fat tissues of mice were collected and incubated with 10% buffered formalin, then dehydrated in an ascending series of ethanol and cleared in xylene. Then, the tissues were embedded in paraffin wax and sliced into 5-μm-thick sections. Hematoxylin and eosin (HE) staining was performed according to the standard protocol. Images were captured with a Zeiss microscope.

### Adipocyte size measurement

The HE-stained sections of epididymal fat tissue prepared above were visualized and adipocyte sizes were measured. In brief, one section of each mouse (six fields per section) was randomly photographed for analysis. The adipocyte sizes were quantified using Image Pro-Plus 6.0 software (Media Cyberneics Inc., Behesda, MD) [23]. Fat cells at image borders were not counted.

### Oil Red O staining

For Oil Red O (Macklin) staining, the liver tissues were frozen and cut into 7-μm-thick sections and stained with fresh Oil Red O. Images were captured with a Zeiss microscope.

### Dihydroethidium staining

Dihydroethidium (DHE) (Invitrogen™), a redox-sensitive, cell-permeable fluorophore, was employed to evaluate hepatocellular reactive oxygen species (ROS) levels in frozen sections of liver tissues. Briefly, 7-μm-thick sections were washed with PBS (pH 7.4) five times and then incubated with the fluorescent probe DHE (2 μM) at 37 °C for 30 min. Finally, images were captured with a fluorescence microscope (Zeiss).

### Alpha-l-fucosidase level in human stool samples

Alpha-l-fucosidase (FUCA) level was analyzed using an ELISA kit (Jianglai, China) for measuring FUCA level in human stool samples according to the manufacturer’s protocol.

### Plasma analysis and hepatic triglyceride measurements

Lipid distribution in plasma lipoprotein fractions was detected using an automatic biochemistry analyzer. Hepatic triglyceride levels were measured using commercial assay kits (Jiancheng Bioengineering) following the manufacturer’s instructions.

### Statistical analysis

All data were represented as mean ± standard error (SEM), and statistical analyses were performed with GraphPad Prism (version 6; GraphPad Software Inc., San Diego, CA) or R (v3.2.2). The differences between two groups were assessed using the two-tailed Student’s t-test. Data sets that involved more than two groups were assessed by one-way analysis of variance (ANOVA) unless indicated otherwise. A value of p < 0.05 was considered to denote statistical significance.

## Results

### Metagenomics analysis showed that HFD altered the gut microbial functional profile

**Fig. 1:**
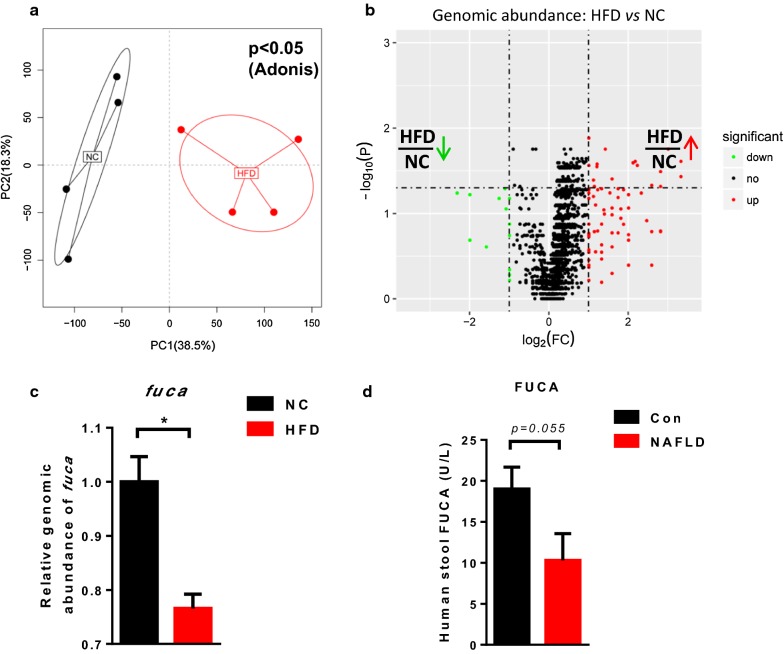
Metagenomic analysis showed that HFD altered the cecal microbial function. **a** Principal coordinate analysis (PCoA) based on metagenomic analysis for cecal content DNA of NC and HFD mice (n = 4). **b** Volcano plot analysing genomic abundance in the HFD group compared with those in the NC group. The x-coordinate is the (log) fold-change (FC) and the y-coordinate is the −log_10_ of the p-value from a t-test (n = 4). **c** Relative genomic abundance of alpha-l-fucosidase (*fuca*) in the cecal content of mice (n = 4). **d** Alpha-l-fucosidase (FUCA) levels in human stool samples (n = 8–9). Data are expressed as mean ± SEM. *p < 0.05, two-tailed Student’s t test, HFD group vs NC group

To characterize the effect of HFD on the gut microbiome, we first performed metagenomics analysis using DNA extracted from cecal contents of mice fed with NC or HFD for 18 weeks. As shown in Fig. 1a, PCoA of the metagenomics data showed a clear separation between NC and HFD clusters (p < 0.05, Adonis analysis). Moreover, volcano plot showed that the abundance of some genes was enriched in the NC group while that of others was enriched in the HFD group (Fig. 1b). We focused on a gene named *fuca*, which had a significantly reduced relative genomic abundance in the HFD group compared with that in the NC group (Fig. 1c) (p < 0.05). To further investigate whether levels of the hydrolase FUCA were altered in stool samples of NAFLD individuals, we measured FUCA levels in samples from healthy controls and NAFLD patients. FUCA levels exhibited a slightly decreasing trend in NAFLD patients, although without statistical significance (Fig. 1d). Taken together, these results suggested that HFD feeding dramatically changed the gut microbial genomics in mice compared with NC feeding.

### Fuc reduced body weight gain and fat mass in HFD-fed mice

**Fig. 2:**
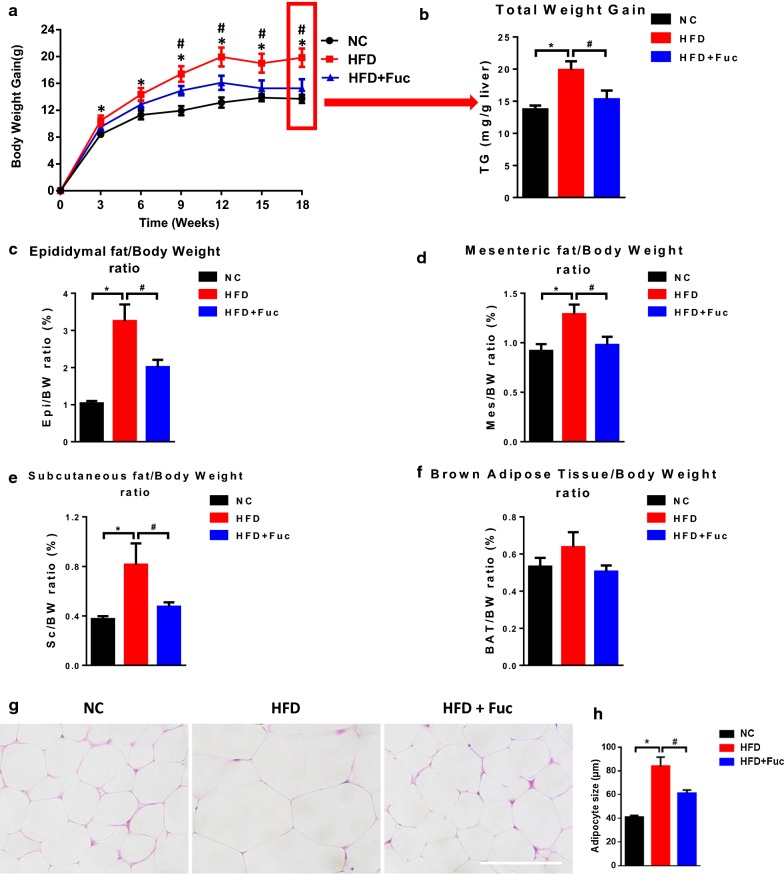
Fuc reduced HFD-induced obesity in mice. **a** Body weight gain. **b** Total body weight gain. **c** The index of epididymal fat (Epi). **d** The index of mesenteric fat (Mes). **e** The index of subcutaneous fat (Sub). **f** The index of brown adipose tissue (BAT). **g** Representative adipocyte size images of HE-stained sections of mouse epididymal fat (Epi) tissue from each group. Scale bar, 100 µm. **h** The adipocyte size quantification. n = 7–12. Data are expressed as mean ± SEM. Difference between groups were assessed by one-way ANOVA. *p < 0.05, HFD group vs NC group; ^**#**^p < 0.05, HFD group vs HFD + Fuc group

The hydrolase FUCA catalytically produces Fuc. To assess whether intragastric administration of Fuc alters the host phenotype upon HFD feeding, we monitored the body weight gain and fat mass in mice. We found that HFD-fed mice gained significantly more weight than NC-fed mice (Fig. 2a). It showed that no difference was found in body weight gain between the HFD group and the HFD + Fuc group before Fuc treatment. Next, Fuc treatment was given to the HFD + Fuc group beginning from the 8th week. Interestingly, compared with the HFD group, the HFD + Fuc group showed reduced body weight with a statistically significant difference at the end of the experiment (Fig. 2b). In addition, the index of epididymal fat (Epi) (Fig. 2c), mesenteric fat (Mes) (Fig. 2d), and subcutaneous fat (Sc) (Fig. 2e) was markedly enhanced in the HFD group compared with the NC group, and the HFD + Fuc group exhibited a significantly lower index than the HFD group indicating an effect of Fuc treatment. Nevertheless, the index of brown adipose tissue (BAT) was moderately elevated in the HFD group but non-significantly compared to the other groups (Fig. 2f). Further, the effect of Fuc treatment on adipocyte size was observed in Epi tissue via HE staining (Fig. 2g). The average adipocyte size was markedly increased in the HFD group compared with the NC group, and decreased in the HFD + Fuc group compared with that in the HFD group (Fig. 2h). Altogether, these results demonstrated that Fuc treatment ameliorated HFD-induced obesity in mice.

### Fuc did not improve host glucose tolerance or insulin tolerance

**Fig. 3:**
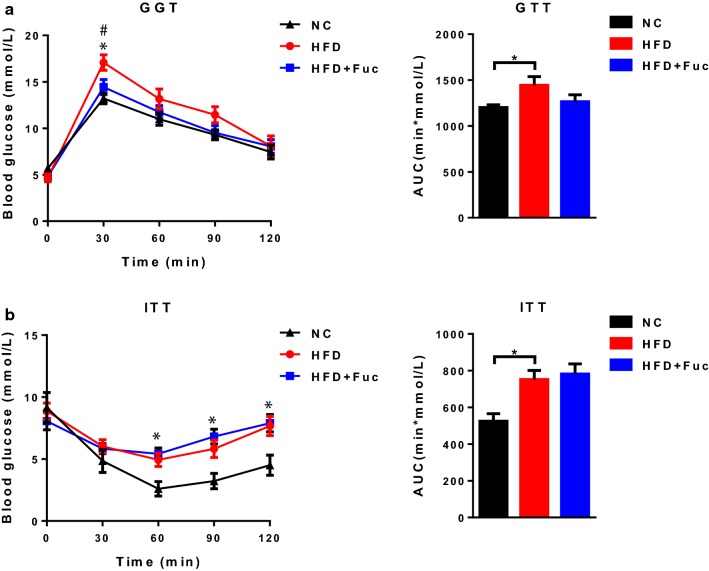
Effects of Fuc on glucose tolerance. **a** Glucose tolerance test (GTT) and the area under curve (AUC) in each group (n = 4–10); **b** Insulin tolerance test (ITT) and the area under curve (AUC) in each group (n = 4–6). Data are expressed as mean ± SEM. Difference between groups were assessed by one-way ANOVA. *p < 0.05, HFD group vs NC group; ^**#**^p < 0.05, HFD group vs HFD + Fuc group

Given that Fuc reduced body weight gain and fat mass (Fig. 2a–e), we next explored whether Fuc treatment ameliorated host glucose metabolism. First, intraperitoneal GTT was used to evaluate glucose tolerance in mice. We found that the area under the curve (AUC) of plasma glucose levels increased in the HFD group after intraperitoneal glucose administration. However, this parameter was moderately but non-significantly affected in the HFD + Fuc group (Fig. 3a). Then, we assessed the insulin tolerance using ITT. As expected, HFD feeding increased the AUC of plasma glucose levels compared with NC. Mice treated with Fuc did not show improved insulin tolerance (Fig. 3b). These results demonstrated that glucose tolerance was impaired in HFD-fed mice. Although Fuc treatment reduced body weight gain and fat mass in HFD-fed mice, it had no significant effect on host glucose metabolism under HFD feeding in current study.

### Fuc ameliorated HFD-induced fatty liver development

**Fig. 4:**
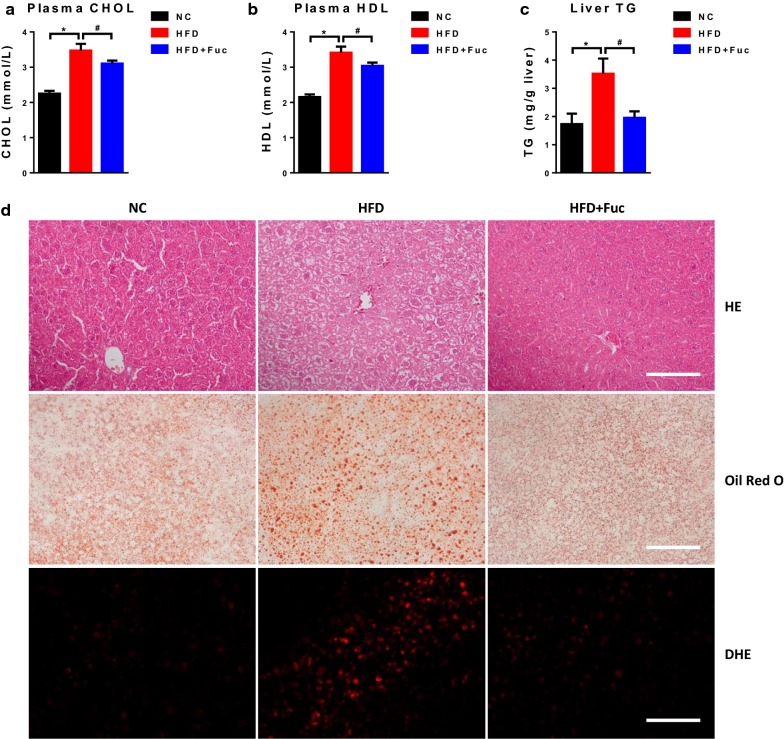
Fuc ameliorated HFD-induced hepatic steatosis in mice. **a** Plasma levels of cholesterol (CHOL). **b** Plasma levels of high-density lipoprotein (HDL). **c** Hepatic triglyceride (TG) level. **d** Representative of HE-stained, Oil Red O-stained, and DHE-stained liver sections. Scale bars = 100 µm. n = 7–12. Data are expressed as mean ± SEM. Difference between groups were assessed by one-way ANOVA. *p < 0.05, HFD group vs NC group; ^**#**^p < 0.05, HFD group vs HFD + Fuc group

Earlier studies have shown an association between HFD and hepatic steatosis in mice [24, 25]. To investigate whether the ameliorating effect of Fuc on hepatic steatosis was associated with Fuc-altered body weight gain, we measured plasma cholesterol (CHOL), plasma high-density lipoprotein (HDL), and hepatic triglyceride (TG) concentration in the mice. We found that plasma concentrations of CHOL and HDL were significantly increased in the HFD group compared with those in the NC group, and they were significantly decreased in the HFD + Fuc group compared with those in the HFD group (Fig. 4a, b). Most notably, hepatic triglyceride levels were markedly increased in the HFD group compared with those in the NC group, and significantly declined following Fuc treatment (Fig. 4c). These data suggested that Fuc treatment ameliorated HFD-induced hepatic steatosis. These findings were further supported by results of liver histology including HE staining, Oil Red O staining, and DHE staining (Fig. 4d). These results collectively indicated that Fuc ameliorated HFD-induced fatty liver development.

### Fuc restored HFD-induced dysbiosis at both compositional and functional levels

**Fig. 5:**
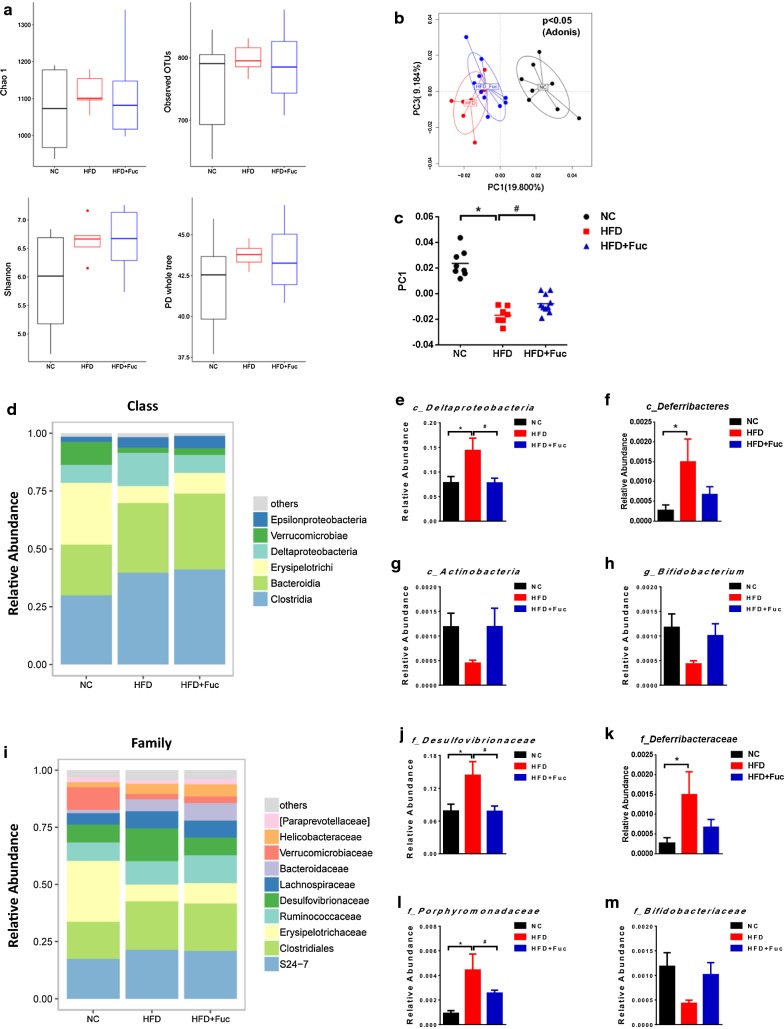
Fuc influenced the composition of cecal microbiota in mice. **a** Alpha diversity indices (Chao 1, observed OTUs, Shannon and PD whole tree) in bacterial microbiomes. **b** Principal coordinate analysis (PCoA) based on the abund jaccard distance analysis of operational taxonomic units (OTUs). **c** The principal component 1 (PC1) distance in abund jaccard distance analysis among the three groups. **d** Relative abundance at the class level of all the three groups. Relative abundance of **e** *Deltaproteobacteria,* **f** *Deferribacteres,* and **g** *Actinobacteria* at the class level. **h** Relative abundance of *Bifidobacterium* at the genus level. **i** Relative abundance at the family level of all the three groups. Relative abundance of **j** *Desulfovibrionaceae,* **k** *Deferribacteraceae,* **l** *Porphyromonadaceae,* and **m** *Bifidobacteriaceae* at the family level. n = 7–12. Data are expressed as mean ± SEM. Difference between groups were assessed by one-way ANOVA. *p < 0.05, HFD group vs NC group; ^**#**^p < 0.05, HFD group vs HFD + Fuc group

Metagenomic analysis of cecal microbiota showed that the relative genomic abundance of the *fuca* gene was reduced in response to HFD (Fig. 1c). Subsequently, we performed 16S rRNA gene sequencing to investigate whether Fuc treatment could normalize the cecal microbiota in HFD-fed mice. We first characterized the composition of cecal microbiota in NC and HFD-fed mice, as well as that in HFD-fed mice treated with Fuc. There were no significant changes in the alpha-diversity of bacteria in terms of Chao 1, observed OTUs, Shannon and phylogenetic diversity (PD) whole tree among the three groups (Fig. 5a). However, PCoA of the abund jaccard uniFrac distance between the cecal contents in each group showed a clear separation between the NC and HFD microbial communities; interestingly, the cecal contents from the HFD + Fuc group were more closely clustered with the microbial communities in the NC group than with those in the HFD group (p < 0.05, Adonis analysis) (Fig. 5b). Specifically, the PC1 distance among the groups exhibited statistical significance (p < 0.05) (Fig. 5c). Analysis at the abundance of class level (Fig. 5d) and family level (Fig. 5i) was presented. At the class level, levels of *Deltaproteobacteria* (Fig. 5e) and *Deferribacteres* (Fig. 5f) were significantly increased and *Actinobacteria* (Fig. 5g) levels were decreased but not significant in the HFD group compared to those in the NC group, but the effect of HFD on *Deltaproteobacteria* was abolished in HFD-fed mice due to Fuc treatment (Fig. 5e). The relative abundance of *Deferribacteres* and *Actinobacteria* in the HFD + Fuc group showed a trend towards that under NC conditions but without significance compared with that in the HFD group (Fig. 5f, g). At the family level, abundance of bacteria belonging to the *Desulfovibrionaceae*, *Deferribacteraceae*, and *Porphyromonadaceae* was significantly increased in response to HFD feeding compared with NC feeding, while Fuc treatment resulted in a decreasing trend in the bacterial levels compared with those in the HFD group (Fig. 5j–l). In particular, both the *Desulfovibrionaceae* (are from the *Deltaproteobacteria* class) and *Porphyromonadaceae* (are from the *Bacteroidia* class) attained statistical significance (Fig. 5j, l). These findings are consistent with those of a previous study by Zhang et al. [26], which found that the *Desulfovibrionaceae* were enhanced and *Bifidobacterium* spp. were absent in mouse models of HFD-induced obesity. Our study also found that bacteria in the *Bifidobacteriaceae* family (Fig. 5m) and the *Bifidobacterium* genus (Fig. 5h) were showed a reducing trend in the HFD group and an increasing trend without significance in the HFD + Fuc group. This indicated that HFD-induced changes in cecal microbiota composition could be reversed due to Fuc treatment.

**Fig. 6:**
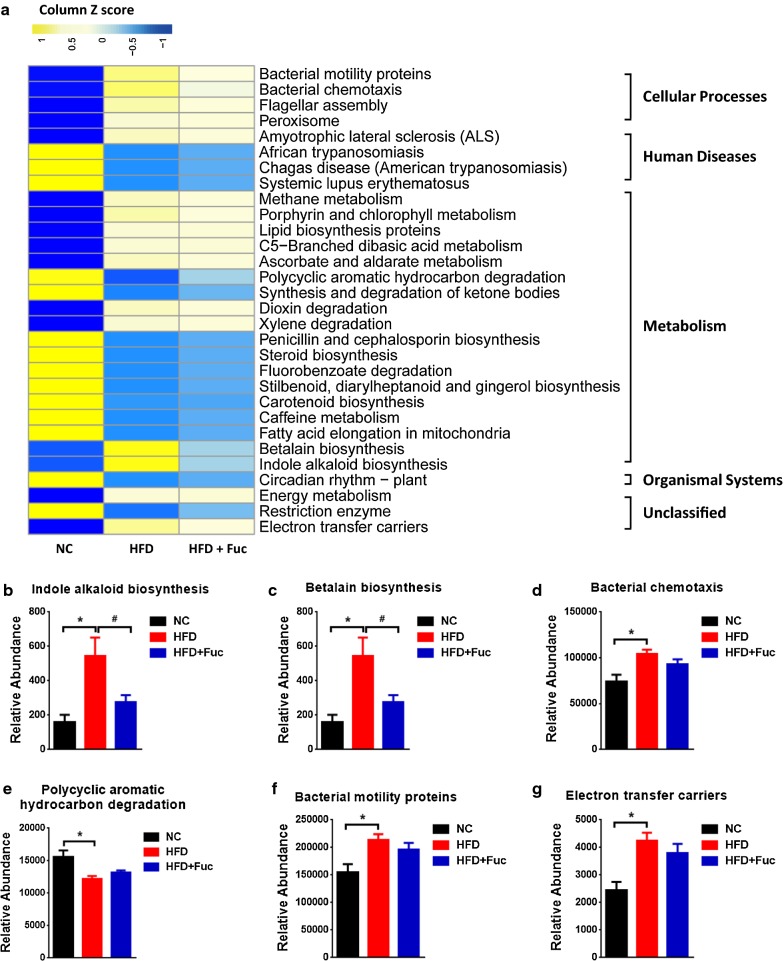
Fuc influenced the function of cecal microbiota. **a** Heatmap showing the main functional pathways with differences among the groups; data were analyzed by Kruskal Waills test. **b**–**g** Representative of functional pathways. n = 7–12. Data are expressed as mean ± SEM. Difference between groups were assessed by one-way ANOVA. *p < 0.05, HFD group vs NC group; ^**#**^p < 0.05, HFD group vs HFD + Fuc group

In addition to the changes in gut microbiota at the compositional level, we next evaluated the cecal microbiota at the functional level using the Phylogenetic Investigation of Communities by Reconstruction of Unobserved States (PICRUSt) analysis. A heatmap was constructed to depict the main functional pathways showing differences among the three groups. All these pathways showed significant differences between the NC group and the HFD group, while Fuc treatment in HFD mice (HFD + Fuc) slightly or modestly reversed the differences (Fig. 6a) (p < 0.05, Kruskal Waills test). These data indicated that the functional pathways altered the most were metabolic pathways. Several representative pathways are shown in Fig. 6b–g. Collectively, these results demonstrated that HFD altered the composition and function of gut microbiota, and treatment with Fuc restored HFD-induced dysbiosis at both compositional and functional levels.

## Discussion

Previous studies have shown that obesity is correlated with a change in intestinal microbiota [27, 28]. However, the mechanisms involved in the response of the microbiota to HFD and the consequent impact on obesity is still unclear. In the present study, based on metagenomic analysis of cecal microbiota, we report that the *fuca* gene is markedly reduced in HFD-fed mice compared with NC-fed mice. Because Fuc production is catalyzed by FUCA, we treated the HFD-fed mice with Fuc via oral gavage to study the relationship among gut microbiota, body weight gain, and hepatic steatosis. We found that Fuc treatment decreased body weight gain, fat accumulation, and hepatic triglyceride elevation in HFD-fed mice for the first time. Most notably, the relative abundance of *Desulfovibrionaceae* was significantly decreased in HFD-fed mice due to Fuc treatment. To our knowledge, the present study is the first to implicate the *fuca* gene and Fuc in the above effects and to demonstrate the beneficial effect of oral Fuc in a mouse model. Fuc is a natural monosaccharide present in mammals, and no traditional toxicology studies on Fuc have been reported previously [29]. Our results provide a novel insight supporting the development of new supplementary strategy of using Fuc treatment to modulate gut microbiota in obese individuals, with potentially positive consequences on body weight loss and relieve of hepatic steatosis.

There are several notable highlights in the present study, but some limitations need to be acknowledged. First, the mechanisms involved in the effect of Fuc on the composition and function of gut microbiota remain largely unknown and need further investigation. In recent years, dysbiosis of the gut microbiota has been suggested to be an important factor in obesity. Many studies have reported that probiotics and prebiotics are used as treatment and preventive measures for multiple human disorders [30]. Beneficial strains including *Lactobacillus* spp. [31, 32], *Akkermansia muciniphila* [33, 34], and *Bifidobacterium* spp. [35, 36] have been reported as potential probiotics which administration is inversely correlated with body weight gain or insulin resistance in rodents and humans. In the present study, switching to a HFD resulted in an increase in *Desulfovibrionaceae*, whereas the abundance of the *Bifidobacterium* genus was showed a decreasing trend, consistent with a previous study showing that levels of the sulphate-reducing and endotoxin-producing *Desulfovibrionaceae* family were enhanced and that the gut barrier-protecting *Bifidobacterium* spp. were absent in HFD-fed mice with the most serious obesity and impaired glucose tolerance phenotypes [26]. We found that levels of the *Desulfovibrionaceae* were significantly decreased and whereas *Bifidobacterium* showed an increasing trend in HFD-fed mice with Fuc treatment. Therefore, the potential effect of Fuc on the above bacteria could also contribute to amelioration of obesity. Future studies are warranted to explore the mechanisms involved.

Second, many studies have reported a relationship between insulin resistance and adipose tissue accumulation. In our study, Fuc reduced fat mass in HFD-fed mice. However, we observed that mice treated with Fuc did not improve glucose tolerance in our GTT (Fig. 3a) or ITT studies (Fig. 3b). The reason why Fuc had no effect on host glucose metabolism under HFD feeding would need further investigation. An alternative explanation is that the lack of effect is related to the dose of Fuc used. The dose of Fuc used in the present study (0.3 g/kg) was based on a previous study which evaluated the effects of Fuc on cell surface glycosylation and tumor cell apoptosis in vivo, and other anti-cancer properties [37]. Future studies are needed to determine whether Fuc administration over a dosage gradient, resulting in increased host exposure, would affect host glucose tolerance.

## Conclusions

In summary, our study demonstrates that Fuc ameliorates obesity and hepatic steatosis in HFD-fed mice. These effects of intragastric administration of Fuc are likely to be due to restoration of HFD-induced enteric dysbiosis at both compositional and functional levels. Notably, levels of the endotoxin-producing bacteria of the *Desulfovibrionaceae* family were decreased in HFD-fed mice treated with Fuc. Fuc is likely to be positioned as a nutraceutical product representing a therapeutic strategy for prevention and treatment of human diseases.

## Abbreviations

AUC: area under the curve
BAT: brown adipose tissue
BW: body weight
CHOL: cholesterol
DHE: dihydroethidium
Epi: epididymal fat
FC: fold-change
Fuc: l-fucose
*fuca*: alpha-l-fucosidase (gene)
FUCA: alpha-l-fucosidase (hydrolase)
GTT: glucose tolerance test
HDL: high-density lipoprotein
HE: hematoxylin and eosin
HFD: high-fat diet
ITT: insulin tolerance test
KEGG: Kyoto Encyclopedia of Genes and Genomes
Mes: mesenteric fat
NAFLD: non-alcoholic fatty liver disease
NASH: non-alcoholic steatohepatitis
NC: normal chow
ANOVA: analysis of variance
OTUs: operational taxonomic units
PBS: phosphate buffer solution
PC: principal component
PCoA: principal coordinate analysis
PCR: polymerase chain reaction
PD: phylogenetic diversity
PICRUSt: Phylogenetic Investigation of Communities by Reconstruction of Unobserved States
ROS: reactive oxygen spesies
Sc: subcutaneous fat
TG: triglyceride
V4: variable region 4

## References

[1] Ogden CL, Yanovski SZ, Carroll MD, Flegal KM (2007). The epidemiology of obesity. Gastroenterology.

[2] Wang FS, Fan JG, Zhang Z, Gao B, Wang HY (2014). The global burden of liver disease: the major impact of China. Hepatology.

[3] Possamai LA, McPhail MJ, Khamri W, Wu B, Concas D, Harrison M (2015). The role of intestinal microbiota in murine models of acetaminophen-induced hepatotoxicity. Liver Int..

[4] Gong S, Lan T, Zeng L, Luo H, Yang X, Li N (2018). Gut microbiota mediates diurnal variation of acetaminophen induced acute liver injury in mice. J Hepatol.

[5] Chen P, Torralba M, Tan J, Embree M, Zengler K, Starkel P (2015). Supplementation of saturated long-chain fatty acids maintains intestinal eubiosis and reduces ethanol-induced liver injury in mice. Gastroenterology.

[6] Chen P, Stärkel P, Turner JR, Ho SB, Schnabl B (2015). Dysbiosis-induced intestinal inflammation activates TNFRI and mediates alcoholic liver disease in mice. Hepatology.

[7] Jiang C, Xie C, Li F, Zhang L, Nichols RG, Krausz KW (2015). Intestinal farnesoid X receptor signaling promotes nonalcoholic fatty liver disease. J Clin Invest..

[8] Le Roy T, Llopis M, Lepage P, Bruneau A, Rabot S, Bevilacqua C (2013). Intestinal microbiota determines development of non-alcoholic fatty liver disease in mice. Gut.

[9] Williams CD, Stengel J, Asike MI, Torres DM, Shaw J, Contreras M (2011). Prevalence of nonalcoholic fatty liver disease and nonalcoholic steatohepatitis among a largely middle-aged population utilizing ultrasound and liver biopsy: a prospective study. Gastroenterology.

[10] Jiao N, Baker SS, Chapa-Rodriguez A, Liu W, Nugent CA, Tsompana M (2018). Suppressed hepatic bile acid signalling despite elevated production of primary and secondary bile acids in NAFLD. Gut.

[11] Chang CJ, Lin CS, Lu CC, Martel J, Ko YF, Ojcius DM (2015). Ganoderma lucidum reduces obesity in mice by modulating the composition of the gut microbiota. Nat Commun..

[12] Porras D, Nistal E, Martínez-Flórez S, Pisonero-Vaquero S, Olcoz JL, Jover R (2017). Protective effect of quercetin on high-fat diet-induced non-alcoholic fatty liver disease in mice is mediated by modulating intestinal microbiota imbalance and related gut-liver axis activation. Free Radic Biol Med..

[13] Smith PM, Howitt MR, Panikov N, Michaud M, Gallini CA, Bohlooly-Y M (2013). The microbial metabolites, short-chain fatty acids, regulate colonic Treg cell homeostasis. Science.

[14] Shull KH, Miller ON (1960). Formation in vivo of glycogen by certain intermediates of the lactate-propanediol pathway. J Biol Chem.

[15] Coffey JW, Miller ON, Sellinger OZ (1964). The metabolism of l-fucose in the rat. J Biol Chem.

[16] Robert L, Fodil-Bourahla I, Bizbiz L, Robert AM (2004). Effect of l-fucose and fucose-rich polysaccharides on elastin biosynthesis, in vivo and in vitro. Biomed Pharmacother.

[17] Fouts DE, Torralba M, Nelson KE, Brenner DA, Schnabl B (2012). Bacterial translocation and changes in the intestinal microbiome in mouse models of liver disease. J Hepatol.

[18] Wang L, Fouts DE, Starkel P, Hartmann P, Chen P, Llorente C (2016). Intestinal REG3 lectins protect against alcoholic steatohepatitis by reducing mucosa-associated microbiota and preventing bacterial translocation. Cell Host Microbe.

[19] KanehisaM Goto S (2000). KEGG: kyoto Encyclopedia of Genes and Genomes. Nucleic Acids Res.

[20] Xiao L, Feng Q, Liang S, Sonne SB, Xia Z, Qiu X (2015). A catalog of the mouse gut metagenome. Nat Biotechnol.

[21] Langille MG, Zaneveld J, Caporaso JG, McDonald D, Knights D, Reyes JA (2013). Predictive functional profiling of microbial communities using 16S rRNA marker gene sequences. Nat Biotechnol.

[22] Singh V, Chassaing B, Zhang L, San Yeoh B, Xiao X, Kumar M (2015). Microbiota-dependent hepatic lipogenesis mediated by stearoyl CoA desaturase 1 (SCD1) promotes metabolic syndrome in TLR5-deficient mice. Cell Metab.

[23] Monickaraj F, Gokulakrishnan K, Prabu P, Sathishkumar C, Anjana RM, Rajkumar JS (2012). Convergence of adipocyte hypertrophy, telomere shortening and hypoadiponectinemia in obese subjects and in patients with type 2 diabetes. Clin Biochem.

[24] Roychowdhury S, McCullough RL, Sanz-Garcia C, Saikia P, Alkhouri N, Matloob A (2016). Receptor interacting protein 3 protects mice from high-fat diet-induced liver injury. Hepatology.

[25] Ma X, Hua J, Li Z (2008). Probiotics improve high fat diet-induced hepatic steatosis and insulin resistance by increasing hepatic NKT cells. J Hepatol.

[26] Zhang C, Zhang M, Wang S, Han R, Cao Y, Hua W (2010). Interactions between gut microbiota, host genetics and diet relevant to development of metabolic syndromes in mice. ISME J.

[27] Ley RE, Turnbaugh PJ, Klein S, Gordon JI (2006). Microbial ecology: human gut microbes associated with obesity. Nature.

[28] Million M, Maraninchi M, Henry M, Armougom F, Richet H, Carrieri P (2012). Obesity-associated gut microbiota is enriched in Lactobacillus reuteri and depleted in *Bifidobacterium animalis* and *Methanobrevibacter smithii*. Int J Obes.

[29] Choi SS, Lynch BS, Baldwin N, Dakoulas EW, Roy S, Moore C (2015). Safety evaluation of the human-identical milk monosaccharide, l-fucose. Regul Toxicol Pharmacol.

[30] Lee SJ, Bose S, Seo JG, Chung WS, Lim CY, Kim H (2014). The effects of co-administration of probiotics with herbal medicine on obesity, metabolic endotoxemia and dysbiosis: a randomized double-blind controlled clinical trial. Clin Nutr.

[31] Bagarolli RA, Tobar N, Oliveira AG, Araújo TG, Carvalho BM, Rocha GZ (2017). Probiotics modulate gut microbiota and improve insulin sensitivity in DIO mice. J Nutr Biochem.

[32] Kim SW, Park KY, Kim B, Kim E, Hyun CK (2013). Lactobacillus rhamnosus GG improves insulin sensitivity and reduces adiposity in high-fat diet-fed mice through enhancement of adiponectin production. Biochem Biophys Res Commun.

[33] Everarda A, Belzer C, Geurts L, Ouwerkerk JP, Druart C, Bindels LB (2013). Cross-talk between *Akkermansia muciniphila* and intestinal epithelium controls diet-induced obesity. Proc Natl Acad Sci USA..

[34] Dao MC, Everard A, Aron-Wisnewsky J, Sokolovska N, Prifti E, Verger EO (2016). *Akkermansia muciniphila* and improved metabolic health during a dietary intervention in obesity: relationship with gut microbiome richness and ecology. Gut.

[35] Chen J, Wang R, Li XF, Wang RL (2012). *Bifidobacterium adolescentis* supplementation ameliorates visceral fat accumulation and insulin sensitivity in an experimental model of the metabolic syndrome. Br J Nutr.

[36] Cano PG, Santacruz A, Trejo FM, Sanz Y (2013). Bifidobacterium CECT 7765 improves metabolic and immunological alterations associated with obesity in high-fat diet-fed mice. Obesity.

[37] Tomsik P, Soukup T, Cermakova E, Micuda S, Niang M, Sucha L (2011). l-rhamnose and l-fucose suppress cancer growth in mice. Open Life Sci.

