# Correction to: l-Fucose ameliorates high-fat diet-induced obesity and hepatic steatosis in mice

**DOI:** 10.1186/s12967-020-02444-3

**Published:** 2020-07-08

**Authors:** Guangyan Wu, Mengwei Niu, Wenli Tang, Jingjuan Hu, Guoquan Wei, Zhanke He, Yangping Chen, Yong Jiang, Peng Chen

**Affiliations:** 1 Department of Pathophysiology, Guangdong Provincial Key Laboratory of Proteomics, School of Basic Medical Sciences, Southern Medical University, Guangzhou, China; 2 State Key Laboratory of Organ Failure Research, Southern Medical University, Guangzhou, China; 3 Division of Laboratory Medicine, Zhujiang Hospital, Southern Medical University, Guangzhou, China

## Correction to: J Transl Med (2018) 16:344 10.1186/s12967-018-1718-x

It was highlighted that the original article [1] contained an error in two figure labels. In Fig. 2b, Y axis label “TG (mg/g liver)” was not correct. The correct label is “Total body weight gain (g)”. In Fig. 3a, the figure head “GGT” was not correct, the correct figure head is: “GTT”.

## References

[1] Wu G, Niu M, Tang W, Hu J, Wei G, He Z, Chen Y, Jiang Y, Chen P (2018). l-Fucose ameliorates high-fat diet-induced obesity and hepatic steatosis in mice. J Transl Med.

